# Cross-cultural adaption and inter-rater reliability of the Swedish version of the updated clinical frailty scale 2.0

**DOI:** 10.1186/s12877-023-04525-6

**Published:** 2023-12-05

**Authors:** Henrik Olsson, Kristina Åhlund, Joakim Alfredsson, David Andersson, Anne-Marie Boström, Susanne Guidetti, Mattias Prytz, Niklas Ekerstad

**Affiliations:** 1 Department of Research and Development, NU Hospital Group, Trollhättan, Sweden; 2 Department of Cardiology, NU Hospital Group, Trollhättan, Sweden; 3 Department of Health Sciences, University West, Trollhättan, Sweden; 4 Department of Cardiology and Department of Health, Medicine and Caring Sciences, Unit of Cardiovascular Sciences, Linköping University, Linköping, Sweden; 5 Department of Management and Engineering, Division of Economics, Linköping University, Linköping, Sweden; 6 Department of Neurobiology, Division of Nursing, Karolinska Institutet, Care Sciences&Society (NVS), Huddinge, Sweden; 7 Karolinska University Hospital, Theme Inflammation and Aging, Stockholm, Sweden; 8 Stockholms Sjukhem, Research and Development Unit, Stockholm, Sweden; 9 Department of Neurobiology, Division of Occupational Therapy, Karolinska Institutet, Care Sciences&Society (NVS), Huddinge, Sweden; 10 Women’s Health and Allied Health Professionals Theme, Medical Unit Occupational Therapy and Physiotherapy, Karolinska University Hospital, Solna, Sweden; 11 Department of Surgery, Institute of Clinical Sciences, Sahlgrenska Academy,, University of Gothenburg, Gothenburg, Sweden; 12 Department of Surgery, NU-Hospital Group, Region Västra Götaland, Trollhättan, Sweden; 13 Department of Health, Medicine, and Caring Sciences, Unit of Health Care Analysis, Linköping University, Linköping, Sweden

**Keywords:** Older adults, Frailty, Clinical frailty scale, ISPOR translation, Validation, Inter-rater reliability

## Abstract

**Background:**

Worldwide, there is a large and growing group of older adults. Frailty is known as an important discriminatory factor for poor outcomes. The Clinical Frailty Scale (CFS) has become a frequently used frailty instrument in different clinical settings and health care sectors, and it has shown good predictive validity. The aims of this study were to describe and validate the translation and cultural adaptation of the CFS into Swedish (CFS-SWE), and to test the inter-rater reliability (IRR) for registered nurses using the CFS-SWE.

**Methods:**

An observational study design was employed. The ISPOR principles were used for the translation, linguistic validation and cultural adaptation of the scale. To test the IRR, 12 participants were asked to rate 10 clinical case vignettes using the CFS-SWE. The IRR was assessed using intraclass correlation and Krippendorff’s alpha agreement coefficient test.

**Results:**

The Clinical Frailty Scale was translated and culturally adapted into Swedish and is presented in its final form. The IRR for all raters, measured by an intraclass correlation test, resulted in an absolute agreement value among the raters of 0.969 (95% CI: 0.929–0.991) and a consistency value of 0.979 (95% CI: 0.953–0.994), which indicates excellent reliability. Krippendorff’s alpha agreement coefficient for all raters was 0.969 (95% CI: 0.917–0.988), indicating near-perfect agreement. The sensitivity of the reliability was examined by separately testing the IRR of the group of specialised registered nurses and non-specialised registered nurses respectively, with consistent and similar results.

**Conclusion:**

The Clinical Frailty Scale was translated, linguistically validated and culturally adapted into Swedish following a well-established standard technique. The IRR was excellent, judged by two established, separately used, reliability tests. The reliability test results did not differ between non-specialised and specialised registered nurses. However, the use of case vignettes might reduce the generalisability of the reliability findings to real-life settings. The CFS has the potential to be a common reference tool, especially when older adults are treated and rehabilitated in different care sectors.

**Supplementary Information:**

The online version contains supplementary material available at 10.1186/s12877-023-04525-6.

## Background

Worldwide, there is a large and growing group of older adults, including patients with complex needs. Frailty is known as an important discriminatory factor for poor outcomes, such as increased dependence, recurrent hospitalisation and death [1–5]. There is no distinct definition of the frailty syndrome, but it is associated with decreased physiological reserves and increased vulnerability to external stressors and is usually described according to either the phenotype model [6] or the accumulated deficit model [7].

The accumulated deficit model is based on the observation that most health risks increase with age and, when a person has more deficits than others of the same age, frailty emerges. A Frailty Index (FI) encompassing 70 different clinical deficits (signs, symptoms, or abnormal test results) [8] constitutes a proxy for biological age [7]. The likelihood of recovering from a vulnerable, albeit non-frail, stage is significantly higher than that from defined frailty, emphasising the importance of early identification and a multi-level assessment tool with discriminatory power [9].

It is important to understand the purpose of the measurement [10, 11]. The assessment of the frailty status and the early identification of frail individuals may lead to appropriate measures, such as a comprehensive geriatric assessment (CGA), to assist in care planning and to reduce disability [12]. The graded assessment of frailty may also provide support in risk prediction and prognostication [7, 13–15]. Furthermore, information on frailty status facilitates the identification of individuals in need of tailored treatments and care plans across health care sectors [13]. This suggests that primary and secondary health care sectors could benefit from the use of a frailty measurement tool with transdisciplinary acceptance [16, 17].

There is no gold standard when it comes to which instrument to use when evaluating frailty in clinical practice. Instead, there are different tools, corresponding to one or more purposes [10, 11, 18]. The use of the FI is regarded as time-consuming and, to promote feasibility in clinical practice, the seven-point Clinical Frailty Scale (CFS-7) was created [7]. The CFS-7 scale has been updated by the instrument owners twice, first to a nine-point scale (CFS-9 1.2) (Canadian Study on Health & Aging, revised 2008) and more recently to CFS-9 2.0 [19].

The CFS has become a frequently used frailty instrument in different clinical settings and health care sectors and it has shown good predictive validity regarding multiple outcomes [20–24]. In the CFS, an older adult’s overall fitness or frailty is graded. It mixes disability, comorbidity and cognitive status. A higher score represents higher risk. The scale focuses on functions in older adults that are considered easy to observe and that do not require a long learning period to measure. This includes mobility, the use of walking aids and the ability to eat, dress and shop etc. The nine-point scale was developed to further improve the grading [19].

### Description of the CFS-9 scale

The CFS-9 1.2 is a scale, on which the first three steps denote different grades of fitness and does not include frailty. The fourth step, *vulnerable,* marks the point at which most people begin to need help. Steps 5 to 8 describe states of progressively increasing frailty and, finally, the ninth step applies to those who are in a state of terminal illness, with short expected survival, but no other signs of frailty. There is a short descriptive text and, optionally, a pictogram associated with each step, in order to facilitate the assessment process and there is also guidance on estimating frailty in older adults with dementia.

In 2020, the CFS-9 was further revised (version 2.0) with minor edits to the level descriptions and their corresponding labels. CFS label 2 changed from “Well” to “Fit”, level 4 from “Vulnerable” to “Living with very mild frailty” and levels 5–8 were restated as “Living with…” mild, moderate, severe and very severe frailty, respectively. The differences between CFS 1.2 and 2.0 can be regarded as be minor [19, 25]. Importantly, in comparison with CFS 1.2, the level headings in CFS 2.0 more clearly indicate that the health care professional should consider the patient’s baseline health state rather than the state of acute illness (e.g. “living with very mild frailty” instead of “vulnerable”) [19].

The CFS-7 was translated into Swedish in 2009 [26], followed by the CFS-9 1.2 in 2017 [24] and the translation process was described in Swedish on the Linköping University website [27]. Tests have indicated that the inter-rater reliability for raters using the Swedish versions is very good [24, 26, 27]. The use of the instrument has successively increased in clinical practice and in registries and trials: for example, since 2020, the CFS has been registered as a mandatory variable in the Swedish Web-System for Enhancement and Development of Evidence-Based Care in Heart Disease Evaluated According to Recommended Therapies (SWEDEHEART) registry [28]. The CFS-9 2.0 was presented by the instrument owners in 2020 [19] and, to be able progressively to develop knowledge of frailty and its use in a clinical setting, translations should be linguistically validated and tested with regard to reliability before being widely used [25, 29].

The aim of this study was first to translate and culturally adapt the CFS into Swedish (CFS-SWE), describing the translation and linguistic validation process, and second to test the inter-rater reliability for registered nurses using the CFS-SWE.

## Methods

### Translation, cross-cultural adaption and linguistic validation process

**Fig. 1:**
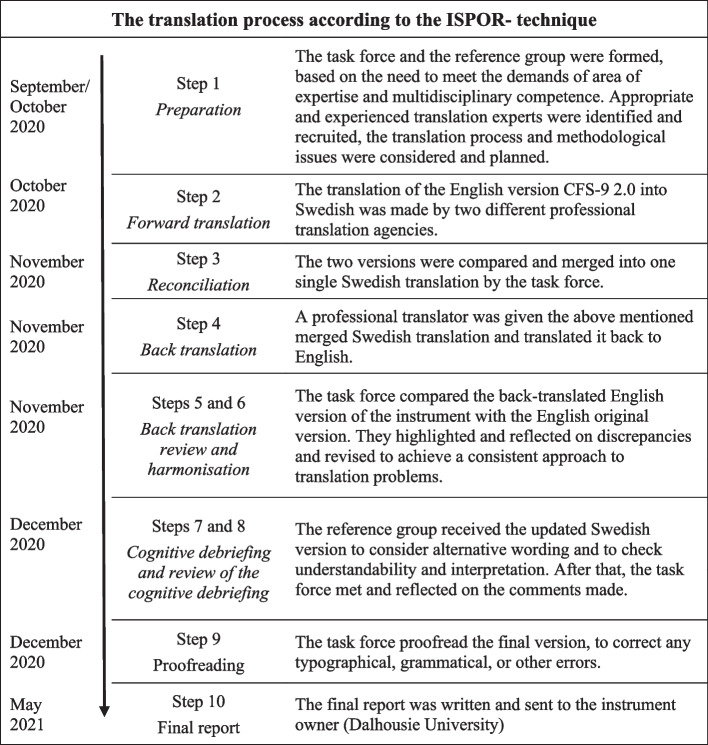
Flowchart of the translation process according to the ISPOR-technique

One of the authors (NE) was asked to take responsibility for the translation process by the CFS copyright holder and instrument developer [7]. The translation and linguistic validation process was conducted from October 2020 to May 2021. To ensure conceptual and cultural compliance with the source instrument [7, 19], the CFS was translated into Swedish using the International Society for Pharmacoeconomics and Outcomes Research (ISPOR) ten-step technique [30], see the flowchart of the translation process in Fig. 1.

The first step, *preparation,* included the initial work before the translation process could start. Relevant co-workers were recruited and a multidisciplinary taskforce was set up. The taskforce consisted of a physician, an RN, a physiotherapist, and an occupational therapist, all of whom had long clinical experience and research merits. A reference group comprising experienced clinicians and researchers in related disciplines and appropriate and experienced expert translators were identified and recruited. Prior to the translation, the process and methodological issues were carefully considered and planned and a brief review of the background to the scale was made, including re-reading and reflection on relevant references [7, 19], previous translations of the CFS-7, CFS-9 1.2 [24, 26] and CFS-9 2.0 [25, 29] instruments and the English language versions of the CFS-9 1.2 and CFS-9 2.0.

The ISPOR process then continued with *forward translation* and *reconciliation* included the translation of the English version of CFS-2.0 (source) into Swedish (target language), by two different professional translation agencies. After that, these two versions were compared and merged into one single Swedish translation by the task force. The next step was *back translation* by a professional translator, in which the above-mentioned merged Swedish translation was translated back to English (back-translated English version).

*The back-translation review* implied that the taskforce compared the back-translated English version of the instrument with the original to highlight and reflect on discrepancies between them. The *harmonisation* step included discussions to achieve a consistent approach to translation problems. After that, the updated Swedish version was tested on relevant reference persons to consider alternative wording and to check understandability, interpretation and cultural relevance, mentioned as the *cognitive debriefing* and *review of the cognitive debriefing step*. The task force then reflected on these comments in relation to the original English version to highlight and amend discrepancies. The ISPOR process ended with *proofreading* of the final version, to correct any typographical, grammatical, or other errors, and a *final report* was written.

### Reliability testing

An estimation of the appropriate numbers of cases and raters in the reliability analyses was made, pragmatically, based on advice from a senior statistician and previously reported reliability tests in similar contexts [24, 27, 29, 31].

Ten case vignettes were produced by two of the authors (NE, KÅ). The case vignettes were built to imitate real-word patients, with influence from ten anonymised cases judged as clinically relevant, and they were selected collectively to represent all nine levels of the CFS. The case vignettes were pragmatically validated by senior clinicians (JA, SG, AMB) with regard to content and CFS grading. The cases provided information on symptoms of disease, diagnoses, dependence on others, cognitive function and the physical status of the patients, see Additional File 1.

The selection of raters was made to identify a sample of study raters, representative of raters handling patients in real-world practice. Since the majority of the CFS grading in a Swedish health care context is undertaken by registered nurses (RNs), the authors recruited twelve raters as a convenience sample from the authors’ network of RNs in the cities of Stockholm, Linköping and Trollhättan (AMB, SG, KÅ). Clinically working RNs, speaking the Swedish language, who accepted participation in the study, were included. Exclusion criteria: other professions than RN, retired RNs, not speaking the Swedish language.

Our study included RNs from different parts of Sweden with experience from both the primary and secondary health care sectors. Moreover, they represented a wide range of length of clinical experience. Half the raters had previous experience of CFS grading (ranging from one grading a week to one grading every six months). Six of the raters were specialised RNs, while six were non-specialised.

The raters participated voluntarily in the study and their scores were treated confidentially. Two weeks prior to the reliability testing, all the raters were introduced to frailty as a concept, the CFS-9 and how CFS grading should be undertaken. This was done by two experienced raters (NE, KÅ) in a one-hour digital education course.

The raters individually assessed each case in written form according to the nine-point CFS-SWE. The cases were presented in random order of severity, i.e. CFS grade, but they were rated in the same order by each rater. The written answers were sent to and treated confidentially by the authors. The raters had the opportunity to obtain additional information on how to grade CFS from a manual on the SWEDEHEART homepage [28].

An observational study design was employed. We have completed the checklists according to the The Strengthening the Reporting of Observational Studies in Epidemiology (STROBE) [32] and the Guidelines for Reporting Reliability and Agreement Studies (GRRAS) [33] statement checklists, and also considered methodological references regarding the reporting of outcome measurement [34, 35].

### Statistical analysis

A data analysis and research plan was registered in the web-based electronic individual study plan for one of the authors (HO) at Linköping University. https://eisp.liu.se/isp/index Analyses of the inter-rater reliability (IRR) associated with the CFS were made using the intraclass correlation (ICC) test [36, 37] and Krippendorff’s alpha agreement coefficient test [38], both with 95% confidence intervals (CI). According to standard practice, the levels of reliability according to the ICC test were defined before the tests as poor (< 0.50), moderate (0.50–0.74), good (0.75–0.90), and excellent (> 0.90) [39]. The levels of agreement according to Krippendorff’s alpha agreement coefficient test are considered as slight (< 0.2), fair (0.2–0.4), moderate (0.4–0.6), substantial (0.6–0.8) and near-perfect (> 0.8) [40].

The sensitivity of the reliability was examined by separately testing the IRR of the group of specialised RNs and non-specialised RNs respectively.

Statistical analysis was performed using SAS software. The statistical significance threshold for all tests was set at *p* < 0.05.

## Results

### Translation, cross-cultural adaption and linguistic validation

**Fig. 2:**
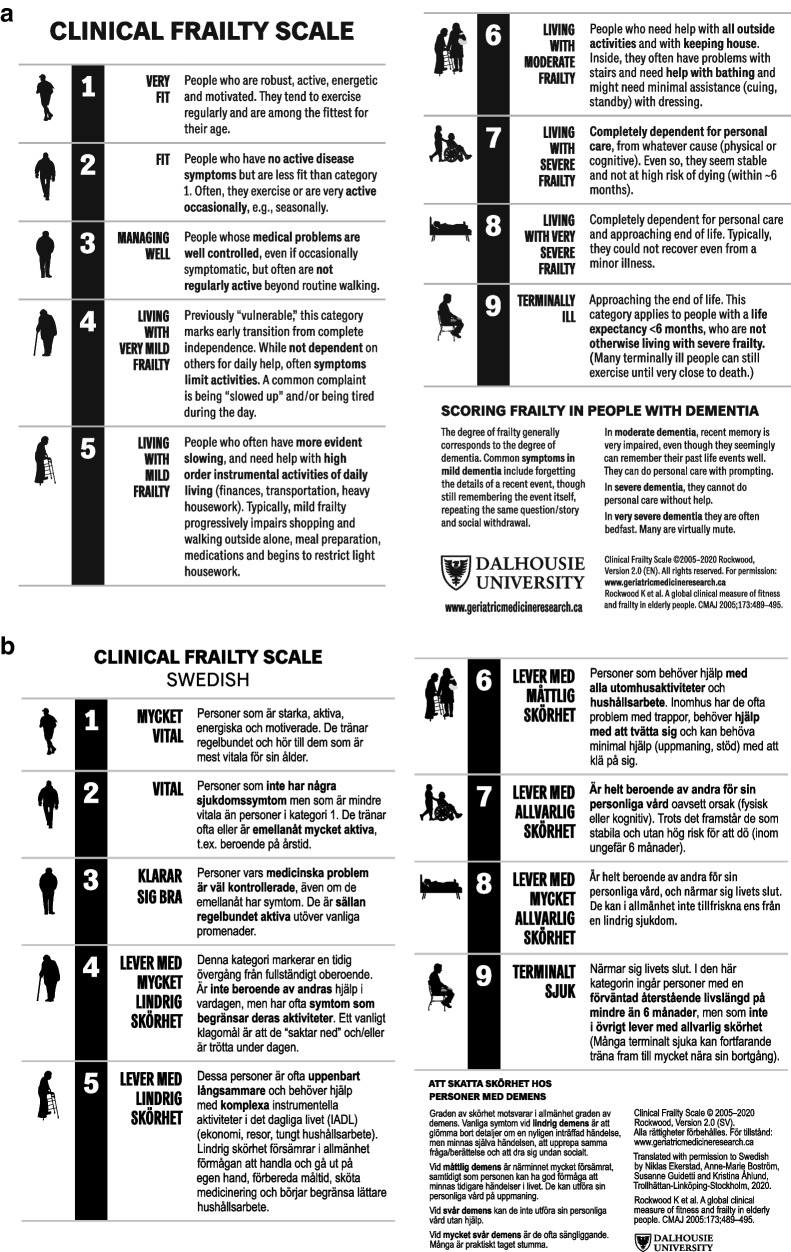
CFS-9 2.0, English (**a**) and Swedish (**b**) versions

The translation and linguistic validation process is shown in Additional File 2, the *final report* in Additional File 3, and the original source instrument (CFS-9 2.0 English) and the Swedish translation (CFS-9 2.0 Swedish) is shown in Fig. 2. Regarding the ISPOR steps of *preparation, forward translation* and *reconciliation*, a high level of agreement in the meaning and wording of the two forward translations was observed. Minor differences were resolved in a reconciliation meeting. The *back translation* step corresponded satisfactorily with the reconciled forward translation. In the *back-translation review and harmonisation step* the task force compared the back-translated English version of the instrument with the English original version. They highlighted and reflected on smaller discrepancies and revised to achieve a consistent approach to translation problems. The steps of *cognitive debriefing* and *review of the cognitive debriefing* indicated good understandability and cultural relevance. In the *proofreading* step, some minor grammatical errors were corrected, Additional File 2, followed by the writing of the *final report*.

### Reliability testing

All 12 raters were RNs and they assessed the 10 cases, yielding 120 observations. The included RNs had a variety of clinical experience from the primary and secondary health care sectors and their length of clinical experience ranged from three to 30 years (median 11.5 yrs). Six of the raters had previous experience of CFS grading (ranging from one grading a week to one grading every six months). Six of the raters were specialised RNs and six of the raters were non-specialised RNs.

The IRR for all raters (based on individual assessments) measured by an ICC test resulted in an absolute agreement value among the raters of 0.969 (95% CI: 0.929–0.991), and a consistency value of 0.979 (95% CI: 0.953–0.994), which indicates excellent reliability. The Krippendorff’s alpha agreement coefficient for all raters was 0.969 (95% CI: 0.917–0.988), indicating near-perfect agreement.

The IRR for non-specialised RNs measured by an ICC test resulted in an absolute agreement value among the raters of 0.948 (95% CI: 0.866–0.985) and a consistency value of 0.969 (95% CI: 0.927–0.991). The Krippendorff’s alpha agreement coefficient for non-specialised RNs was 0.948 (95% CI: 0.857–0.982).

The IRR for specialised RNs measured by an ICC test resulted in an absolute agreement value among the raters of 0.987 (95% CI: 0.970–0.996) and a consistency value of 0.988 (95% CI: 0.927–0.997). The Krippendorff’s alpha agreement coefficient for non-specialised RNs was 0.987 (95% CI: 0.952–0.997).

## Discussion

We systematically translated and validated the CFS into Swedish (CFS-SWE). The translation and linguistic validation was undertaken using the established and recommended ISPOR technique. The translation work was carried out by individuals representing different professions, in order appropriately to address the multidisciplinary components of the CFS. Our analyses showed that the inter-rater reliability for all raters (based on individual assessments) was excellent, according both to the intraclass correlation test and to the Krippendorff’s alpha agreement coefficient test. The reliability test results did not differ between non-specialised and specialised RNs.

These findings agree with the results of translations of previous versions (CFS-7 and CFS-9 1.2 respectively) of the CFS into Swedish [24, 26]. Similarly, translations and validations into other languages have been successfully undertaken using the same technique [25, 29, 31].

The large majority of the CFS gradings in a Swedish health-care context are undertaken by RNs. Our study included RNs from different parts of Sweden with experience from both the primary and secondary health care sectors. Moreover, they represented a wide range of length of clinical experience. Half the raters had previous experience of CFS grading (ranging from one grading a week to one grading every six months). Six of the raters were specialised RNs, while six were non-specialised. We believe that the characteristics of the selected sample of study raters were reasonably representative of CFS-raters handling patients in real-world practice, which indicates good generalisability. This is important, as the assessment according to the CFS is under continuous dispersion in Swedish healthcare, e.g. in the SWEDEHEART and intensive care registries, as well as in clinical practice.

These findings indicate that the CFS-SWE is a reliable and useful measurement of frailty that can be applied and carefully spread in both the primary and secondary health care sectors. This conclusion agrees with the results in previous studies of the translation of the CFS into other languages [25, 29, 31]. Moreover, evidence has been provided of the ability of the scale to predict a range of adverse health outcomes, especially mortality [15, 20–24, 41–44]. Previous studies have indicated that the CFS has good sensitivity, specificity and predictive validity [7].

A frailty assessment is recommended in different guidelines, both geriatric and non-geriatric, e.g. regarding cardiovascular care [11, 18, 44]. It is suggested that a valid, reliable and relatively easily applied measurement of frailty has the potential to improve collaboration in the diagnostics, care, treatment and rehabilitation of frail patients both within and across health-care sectors, such as hospital care, primary care and municipal and social care [29]. This has clinical implications, as doctors, RNs, physiotherapists and occupational therapists could be supported by a frailty measurement when it comes to determining treatment options and planning hospital discharge and rehabilitation. Moreover, a standardised measurement of frailty might assist community RNs in identifying individuals requiring an extra follow-up after hospital care.

In spite of the results of our study, reliability evaluations should be interpreted with caution as they are dependent on assessment conditions [33]. Other properties of the CFS-SWE, such as responsiveness and predictive validity, should be tested in a clinical context more similar to daily practice [24, 29, 43]. Before using the CFS in clinical practice, users should be appropriately introduced to the way CFS grading should be performed.

### Strengths and limitations

This study has several strengths. First, the translation and linguistic validation was completed using a rigorous procedure that followed the ISPOR guidelines. This is important, as quality depends on the chosen methodology and poorly translated instruments threaten the validity of the data [30]. Second, the translation work was carried out by individuals representing different professions, in order appropriately to address the multidisciplinary components of the CFS. Third, the reliability analyses were performed using two types of established reliability test, both indicating excellent reliability. Fourth, the study included key actors most often involved in frailty grading in clinical practice and the characteristics of the selected sample of study raters were reasonably representative of CFS raters handling patients in real-world practice, which indicates good generalisability.

Our study has some limitations. First, case vignettes could be regarded as hypothetical and they might be associated with a risk of bias and inflated inter-rater reliability which has been mentioned in previous studies with similar purpose, e.g. [29]. On the other hand, the method of testing reliability using case vignettes is an established technique [29, 45, 46]. In order to optimise the generalisability of the results of our study, the case vignettes were constructed to reflect real-world patients in our daily clinical practice, and to represent all nine levels of the CFS. Prior to completing the questionnaire, the study raters were given a short introduction to CFS rating. Importantly, the cases were presented in random order of severity, i.e. CFS grade, without guiding pictograms. Cases were individually rated in the same order by each rater.

Second, raters were recruited as a convenience sample, which inherently poses a risk of inflated reliability measurements due to non-random selection. However, we believe that the characteristics of the selected sample of study raters were reasonably representative of CFS raters handling patients in real-world practice, which indicates good generalisability. Third, the number of cases was relatively small (*n* = 10). However, the cases were designed to encompass all the CFS levels, presented in random order of severity, and they had been pragmatically validated by senior raters. Overall, a comparison between studies on the reliability of CFS rating based on clinical case vignettes (e.g. Nissen 2020 [29]  = “excellent”, Young 2020 [45]  = “broad agreement”, Fehlmann 2023 [46]  = “good” and our study = “excellent”) with studies on the reliability of CFS rating of real-world patients (e.g. Abraham 2019 [31]  = “good to excellent”, Vrettos 2021 [47]  = “good”, Flaatten 2021 [48]  = “high to very high”) indicates that the degree of reliability tends to be slightly attenuated with real-world cases. Nevertheless, also in studies on reliability of CFS rating of real-world patients, the reliability was reported to be “good to excellent”. Taken together we believe that our results reflect assessment of real-world patients.

## Conclusions

The Clinical Frailty Scale was translated, linguistically validated and culturally adapted into Swedish following a rigorous and well-established standard technique. The inter-rater reliability for all raters was excellent, judged by two established, separately used, reliability tests. However, the use of case vignettes might reduce the generalisability of the reliability findings to real-life settings. The CFS has the potential to be a common reference tool, especially when older adults are treated and rehabilitated in different care sectors.

### Supplementary Information

**Additional file 1.** Case vignettes in English (translated version).

**Additional file 2. **The translation process.

**Additional file 3.** Final report on the translation process.

**Additional file 4. **GRRAS checklist.

**Additional file 5. **STROBE checklist.

## Data Availability

The datasets used and/or analysed during the current study are available from the corresponding author on reasonable request.

## Abbreviations

CFS: Clinical Frailty Scale
CFS-SWE: Clinical Frailty Scale – Swedish version
CGA: Comprehensive geriatric assessment
CI: Confidence Interval
FI: Frailty Index
IRR: Inter-rater reliability
ICC: Intraclass correlation
ISPOR: International Society for Pharmacoeconomics and Outcomes Research
RN: Registered nurse
SWEDEHEART: Swedish Web-System for Enhancement and Development of Evidence-Based Care in Heart Disease Evaluated According to Recommended Therapies
