# Supplementary material for: Cross-cultural adaption and inter-rater reliability of the Swedish version of the updated clinical frailty scale 2.0
Source: BMC Geriatr. 2023 Dec 5;23:803. doi: 10.1186/s12877-023-04525-6 (PMC10696827; doi:10.1186/s12877-023-04525-6)
Supplement: Supplementary file 1 — Additional file 1. Case vignettes in English (translated version). [file 12877_2023_4525_MOESM1_ESM.docx]

**Additional File 1. Case vignettes in English (translated version)**

**Case 1: Bengt 79 years**

Bengt lives with his wife in a detached house with stairs both indoors and outdoors. No home care service. Performs activities of daily living (ADL) independently. An active and social life with many friends and he usually travels abroad for parts of the winter season.

Chronic atrial fibrillation, treated with anticoagulants (Warfarin) for the past ten years. Ischaemic heart disease with mild and infrequent angina pectoris. Mild heart failure. Benign prostate enlargement.

In the last six months, Bengt has been experiencing symptoms in the form of shortness of breath and fatigue when exerting himself, for example, when climbing stairs.

CFS___________________________________________________________

**Case 2: Greta 77 years**

Greta is a widow and lives alone. Smoker. Walks with the support of a walker outdoors. She carries out her personal ADL independently, but her daughter helps her with bills and purchases.

Hypertension and tablet-treated diabetes. Mild-moderate COPD. Chronic atrial fibrillation, treated with anticoagulants (NOAC).

For about a year, she has been experiencing shortness of breath and fatigue during exertion and dizziness when changing positions.

CFS___________________________________________________________

**Case 3: Berta 92 years**

Berta is a widow. She has been living alone for several years. Lives in her own house with home care service twice a day. Partially dependent in personal ADL, such as going to the toilet, dressing and undressing and showering. Her son helps her with purchases and finances.

Hypertension and tablet-treated diabetes. Moderate heart failure and moderate renal impairment. Repeated urinary tract infections. A month ago, she was treated in hospital due to leg ulcers and sepsis. Persistent fatigue.

CFS___________________________________________________________

**Case 4: Sven-Åke 72 years**

Sven-Åke is married and lives with his wife in a detached house. Former heavy smoker and moderate overconsumption of alcohol.

Hypertension. Mild benign prostatic enlargement. For the past six months, she has had recurring abdominal pain, as well as weight loss and nausea. Three months ago, he was diagnosed with metastatic pancreatic cancer, incurable. He is enrolled with the hospital’s palliative team with home care service and the possibility of hospitalisation when needed. Sven-Åke wants to stay at home for as long as possible, but in recent weeks he has been dealing with an increasingly deteriorating general condition.

CFS___________________________________________________________

**Case 5: Svetlana 74 years**

Svetlana lives alone in an apartment on the fourth floor without an elevator. Divorced. Two children who live nearby. She has a rich social life and hangs out a lot with friends. Performs her ADLs independently. Walks half a mile almost every day and exercises in the gym with a senior group three times a week.

Well-regulated hypertension and well-treated hypothyroidism.

Feels healthy and energetic.

CFS___________________________________________________________

**Case 6: Ros-Marie 81 years**

Ros-Marie has been living in a nursing home for the past two years. Uses a wheelchair and is completely dependent when performing ADLs, including eating. Can only say single words.

Mb Alzheimer’s for a decade, probably also elements of vascular dementia after several strokes, with sequelae in the form of partial right-sided paralysis. Ischaemic heart disease with mild angina. Hypertension and hypercholesterolemia. Moderately impaired renal function.

For the last couple of months, Ros-Marie has been half-asleep most days and is difficult to contact.

CFS___________________________________________________________

**Case 7: Stina 82 years**

Stina lives with her husband in a terraced house. Walks with the support of a walker. Home care service three times a day. Her children help her with bills, finances and medicines. Her husband takes care of the household and helps her to dress and undress. The home care staff help with other ADL, such as going to the toilet, hygiene and bathing.

Insulin-treated diabetes. Hypertension and hypercholesterolemia. Previously a myocardial infarction. Dementia of a moderate degree. Osteoarthritis in the knees and hips.

Stina is often tired during the day, but her condition has been unchanged for the past six months.

CFS___________________________________________________________

**Case 8: Roger 75 years**

Roger lives with his wife in a detached house. An active social life and hangs out a lot with friends. Plays bridge once a week. Performs ADL independently. He takes walks (20-30 minutes) three or four times a week.

Well-treated hypertension. Experiencing no symptoms.

CFS___________________________________________________________

**Case 9: Karl 95 years**

Karl is a widower living alone. No home care service. In the past year, he appears to have become slightly forgetful. His son helps him with bills and purchases. Otherwise, Karl takes care of himself at home and does not need help with personal ADL. Former elite level wrestler. He walks every day and engages in veteran-dancing once a week.

Chronic atrial fibrillation, treated with anticoagulants (NOAC). Benign prostate enlargement with mild discomfort. Mild osteoarthritis in the hips.

CFS___________________________________________________________

**Case 10: Josef 71 years**

Josef lives with his wife in a detached house. Performs daily activities independently. No home care service. He walks with his wife and exercises periodically at the gym (senior pass).

Diet-treated well-regulated diabetes. Well-controlled hypertension. Experiencing no symptoms.

CFS___________________________________________________________
