# Supplementary material for: Cross-cultural adaption and inter-rater reliability of the Swedish version of the updated clinical frailty scale 2.0
Source: BMC Geriatr. 2023 Dec 5;23:803. doi: 10.1186/s12877-023-04525-6 (PMC10696827; doi:10.1186/s12877-023-04525-6)
Supplement: Supplementary file 2 — Additional file 2. The translation process. [file 12877_2023_4525_MOESM2_ESM.docx]

|  | CFS-9 2.0 ENG | CFS-9 2.0 SWE  (final version) | ISPOR- step | Discussions of discrepancies and changes during the process |
| --- | --- | --- | --- | --- |
| 1 | **Very fit** – People who are robust, active, energetic, and motivated. They tend to exercise regularly and are among the fittest for their age. | **Mycket vital** – Personer som är starka, aktiva, energiska och motiverade. De tränar regelbundet och hör till dem som är mest vitala för sin ålder. | **5 and 6** *Back-translation review and harmonisation* | In the first translation, the term *individuals* was used. Agree to consistently change from *individuals* to *persons* in steps 1 to 9. There were also discussions regarding the meaning and differences in the terms *robust* vs. *strong* in Swedish. Retain *strong*. |
|  |  |  | **7 and 8** *Cognitive debriefing and review of the cognitive debriefing* | Consideration according to the reference group comments on the words *fit* vs. *vital,* where there appears to be a certain difference in meaning. However, in relation to frailty, *vital* is considered best suited.  There were also discussions regarding *often exercises regularly*, which is a difficult sentence in Swedish. Agree to delete the word *often*. |
| 2 | **Fit** – People who **have no active disease symptoms** but are less fit than category 1. They often exercise or are very **active occasionally**, e.g., seasonally. | **Vital** – Personer som **inte har några sjukdomssymtom**, men som är mindre vitala än personer i kategori 1. De tränar ofta, eller är **emellanåt mycket aktiva**, t.ex. beroende på årstid. | **5 and 6** *Back-translation review and harmonisation* | No changes |
|  |  |  | **7 and 8** *Cognitive debriefing and review of the cognitive debriefing* | Consideration according to the reference group comments on the Swedish words *active vs. ongoing* and *occasionally vs. sometimes*. The task force chooses not to change any of them. |
| 3 | **Managing well** – People whose **medical problems are well controlled**, albeit occasionally symptomatic, but are **not often regularly active** beyond routine walking. | Klarar sig bra – Personer **vars medicinska problem är väl kontrollerade**, även om de emellanåt har symtom. De är **sällan regelbundet aktiva** utöver vanliga promenader | **5 and 6** *Back-translation review and harmonisation* | No changes |
|  |  |  | **7 and 8** *Cognitive debriefing and review of the cognitive debriefing* | No changes |
| 4 | **Living with very mild frailty** – Previously “vulnerable”, this category marks early transitions from complete independence. While **not dependent** on others for daily help, **symptoms often limit activities**. A common complaint is being “slowed up” and/or being tired during the day. | **Lever med mycket lindrig skörhet** – Denna kategori markerar en tidig övergång från fullständigt oberoende. Är **inte beroende av andras hjälp** i vardagen, men har **ofta symtom som begränsar deras aktiviteter**. Ett vanligt klagomål är att de ”saktar ner” och/eller är trötta under dagen. | **5 and 6** *Back-translation review and harmonisation* | Discussions about the meaning and use of the term *living with* in Swedish. Agree to keep it consistent in steps 4 to 8. |
|  |  |  | **7 and 8** *Cognitive debriefing and review of the cognitive debriefing* | Consideration according to the reference group comments on the word *mark*, which in Swedish mean both *mark* and *distinguish*. Retains mark. |
| 5 | **Living with mild frailty** – People who often have **more evident slowing** and need help with **high order** instrumental activities of daily living (finances, transportation, heavy housework). Typically, mild frailty progressively impairs shopping and walking outside alone, meal preparation and medications and begins to restrict light housework. | **Lever med lindrig skörhet** – Dessa personer är ofta **uppenbart långsammare** och behöver hjälp med **komplexa** instrumentella aktiviteter i det dagliga livet (IADL) (ekonomi, resor, tungt hushållsarbete). Lindrig skörhet försämrar i allmänhet förmågan att handla och gå ut på egen hand, förbereda måltid, sköta medicinering och börjar begränsa lättare hushållsarbete | **5 and 6** *Back-translation review and harmonisation* | Discussion about the meaning and use of the words *high order* vs. *complex* IADL, *transportation* vs. *transport* and *meal* *preparation* vs. *cook.* Agree to keep all of them unchanged |
|  |  |  | **7 and 8** *Cognitive debriefing and review of the cognitive debriefing* | Consideration according to the reference group comments on the words *instrumental* and *progressively impairs*. As IADL is an accepted expression in the field of elderly people, it remains unchanged. In Swedish, there is no exact simple translation of *progressively impair*. Remains unchanged.  The reference group also considered the meaning and use of the words *transportation* (*to transport oneself*) and *travel*. As IADL appears to refer to travel, public transport, driving etc., the task force agrees to change to travel. |
| 6 | **Living with moderate frailty** – People who need help **with all outside activities** and with **keeping house**. Inside, they often have problems with stairs and need **help with bathing** and might need minimal assistance (cuing, standby) with dressing. | **Lever med måttlig skörhet** – personer som behöver hjälp **med alla utomhusaktiviteter** och **hushållsarbete**. Inomhus har de ofta problem med trappor, behöver **hjälp med att tvätta sig** och kan behöva minimal hjälp (uppmaning, stöd) med att klä på sig. | **5 and 6** *Back translation review and harmonisation* | Discussion about the meaning and use of the terms *cuing* vs. *encouragement*. Retain *cuing*. |
|  |  |  | **7 and 8** *Cognitive debriefing and review of the cognitive debriefing* | Consideration according to the reference group comments on the meaning of the word *bathing* and the Swedish word *tvätta sig*. Remains unchanged |
| 7 | **Living with severe frailty** – **Completely dependent for personal care**, from whatever cause (physical or cognitive). Even so, they seem stable and not at high risk of dying (within ~6 months) | **Lever med allvarlig skörhet** – **Är helt beroende av andra för sin personliga vård** oavsett orsak (fysisk eller kognitiv). Trots det framstår de som stabila och utan hög risk att dö (inom ungefär 6 månader). | **5 and 6** *Back translation review and harmonisation* | No changes |
|  |  |  | **7 and 8** *Cognitive debriefing and review of the cognitive debriefing* | No changes |
|  |  |  | **9**  *Proofreading* | The task force adds the Swedish word *sin* (personal care) as a linguistic adaptation, to harmonise with the wording in step 8 and in the dementia sections. |
| 8 | **Living with very severe frailty** – Completely dependent for personal care and approaching end of life. Typically, they could not recover even for a minor illness. | **Lever med mycket allvarlig skörhet** – Är helt beroende av andra för sin personliga vård, och närmar sig livets slut. De kan i allmänhet inte tillfriskna ens från en lindrig sjukdom. | **5 and 6** *Back translation review and harmonization* | Discussion about the formulation *completely dependent for personal care.* Agree to change to *completely dependent on others for personal care*. |
|  |  |  | **7 and 8** *Cognitive debriefing and review of the cognitive debriefing* | No changes |
|  |  |  | **9**  *Proofreading* | The task force adds the Swedish word *är* (completely dependent) as a linguistic adaptation, to harmonize with the wording in step 7. |
| 9 | **Terminally ill** – Approaching the end of life. This category applies to people with a **life expectancy of <6 months**, who are **not otherwise** living with severe frailty. (Many terminally ill people can still exercise until very close to death) | **Terminalt sjuk** – Närmar sig livets slut. I den här kategorin ingår personer med en **förväntad återstående livslängd på mindre än 6 månader**, men som **inte i övrigt lever med alvarlig skörhet**. (Många terminalt sjuka kan fortfarande träna fram till mycket nära sin bortgång). | **5 and 6** *Back translation review and harmonisation* | No changes |
|  |  |  | **7 and 8** *Cognitive debriefing and review of the cognitive debriefing* | Consideration according to the reference group comments on the meaning of the word *death* and the Swedish word *bortgång*. Remains unchanged. |
|  | **Scoring frailty in people  with dementia** | **Att skatta skörhet hos personer  med demens** | **5 and 6** *Back translation review and harmonisation* | Agree consistently to use *persons and not individuals,* even in the dementia section. |
|  |  |  | **7 and 8** *Cognitive debriefing and review of the cognitive debriefing* | No changes |
| I | The degree of frailty generally corresponds to the degree of dementia. Common symptoms in **mild dementia** include forgetting the details of a recent event, despite still remembering the event itself, repeating the same question/story and social withdrawal. | Graden av skörhet motsvarar i allmänhet graden av demens. Vanliga symtom vid **lindrig demens** är att glömma bort detaljer om en nyligen inträffad händelse, men att minnas själva händelsen, att upprepa samma fråga/berättelse och att dra sig undan socialt. | **5 and 6** *Back translation review and harmonisation* | No changes |
|  |  |  | **7 and 8** *Cognitive debriefing and review of the cognitive debriefing* | Consideration according to the reference group comments on the meaning of the words *event* and *story/question.* Remains unchanged. |
| II | **In moderate dementia**, recent memory is very impaired, even though they can remember their past life events well. They can perform personal care with prompting. | Vid **måttlig demens** är närminnet mycket försämrat, samtidigt som personen kan ha god förmåga att minnas tidigare händelser i livet. De kan utföra personlig vård på uppmaning. | **5 and 6** *Back translation review and harmonisation* | Discussions of the meaning of and differences relating to the words *prompting* vs. *if encouraged*. Retains *prompting*.  The two initial translations differ on *these individuals* vs. *they*. Consistently, in the dementia section, the task force chooses *they*. |
|  |  |  | **7 and 8** *Cognitive debriefing and review of the cognitive debriefing* | Consideration according to the reference group comments on the meaning of the Swedish words *tycks ha* vs. *kan ha* (may). The task force agrees to change to *kan ha*. |
| III | **In severe dementia,** they are unable to perform personal care without help. | Vid **svår demens** kan de inte utföra sin personliga vård utan hjälp | **5 and 6** *Back translation review and harmonisation* | No changes |
|  |  |  | **7 and 8** *Cognitive debriefing and review of the cognitive debriefing* | No changes |
| IV | **In very severe dementia,** they are often bedbound. Many are virtually mute. | Vid **mycket svår demens** är de ofta sängliggande. Många är praktiskt taget stumma. | **5 and 6** *Back translation review and harmonisation* | Discussions of the meaning of and differences relating to the words *mute* and the Swedish word *stum*. Finds nothing more appropriate and chooses to keep mute. |
|  |  |  | **7 and 8** *Cognitive debriefing and review of the cognitive debriefing* | No changes |
