# Supplementary material for: Cross-cultural adaption and inter-rater reliability of the Swedish version of the updated clinical frailty scale 2.0
Source: BMC Geriatr. 2023 Dec 5;23:803. doi: 10.1186/s12877-023-04525-6 (PMC10696827; doi:10.1186/s12877-023-04525-6)
Supplement: Supplementary file 3 — Additional file 3. Final report on the translation process. [file 12877_2023_4525_MOESM3_ESM.docx]

**Additional File 3 Final report on the translation process.**

**Process documentation regarding the translation of Clinical Frailty Scale-9, version 2.0 (CFS.EN2.0) into Swedish (CFS.SV2.0). The ISPOR translation technique was used (1).**

**Task force:**

Niklas Ekerstad (project manager), MD, PhD, specialist in internal medicine and cardiology, associate professor, Linköping university, Linköping, and NU Hospital Group, Trollhättan

Anne-Marie Boström, registered nurse, PhD, associate professor, Karolinska institutet, Stockholm

Susanne Guidetti, occupational therapist, PhD, professor, Karolinska institutet, Stockholm

Kristina Åhlund, physiotherapist, PhD, NU Hospital Group and University West, Trollhättan

**Reference group:**

Joakim Alfredsson, MD, PhD, specialist in internal medicine and cardiology, associate professor, Linköping university, Linköping

Jüri Kartus, MD, PhD, specialist in orthopedics, professor, Gothenburg university, Gothenburg

Ninni Sernert, physiotherapist, PhD, professor, Gothenburg university, Gothenburg

**Translators:**

Space 360, translation agency

Proper English AB, translation agency

Jeanette Kliger, professional translator

**Translation period:**

From October 26, 2020 until December 20, 2020

**Sites of translation involved:**

Linköping, Stockholm and Trollhättan

**Steps in the process of translation in accordance with the ISPOR technique (ISPOR steps in parentheses):**

1. **Preparation (1).** Initial work was carried out before the translation began. The task force and the reference group were formed. Selection of members was based on the need to meet demands of area of expertise and multidisciplinary competence. Appropriate and experienced expert translators were identified and recruited. The process of translation was planned and methodological issues were considered. A short review was made concerning the background of the scale including re-reading of relevant references. e.g. (2, 3). Experiences from previous translations of CFS-7.EN and CFS-9.EN1.2 into Swedish versions were reflected on. CFS.EN2.0 was compared with CFS.EN1.2.
2. **Forward translation (2).** Translations of CFS.EN2.0 (source) into Swedish (target language). This was performed independently by two professional translation agencies. Space 360 (October 27, 2020) and Proper English AB (October 31, 2020).
3. **Reconciliation (3)**. The above-mentioned forward translations were compared and merged into one single Swedish translation. This was done via a Zoom meeting with the taskforce November 11, 2020.
4. **Back translation (4).** Translation of the merged Swedish forward translated version back into English (back-translated English version) was done by Jeanette Kliger, November 18, 2020.
5. **Back translation review and harmonisation (5-6).** Comparison of the back-translated English version of the instrument with the original in order to highlight and reflect on discrepancies between the original- and the reconciled translations, which then was revised. Harmonisation in order to achieve a consistent approach to translation problems. This was done in a Zoom meeting with the taskforce November 27, 2020.
6. **Cognitive debriefing and review of the cognitive debriefing (7-8).** The updated Swedish translation was tested on relevant reference persons in order to consider alternative wording and to check understandability and interpretation. Reflection on the reference persons’ comments on this version in relation to the original English version to highlight and amend discrepancies. This was done in a Zoom meeting with the taskforce, December 8, 2020.
7. **Proofreading (9)**. Final review of the translation to highlight and correct any typographic, grammatical or other errors. Finalization of the translation process, December 14, 2020.
8. **Final Report (10).** Report written at the end of the process.

**References:**

1. Wild D, Grove A, Martin M, et al. Principles of Good Practice for the Translation and Cultural Adaptation Process for Patient-Reported Outcomes (PRO) Measures: report of the ISPOR Task Force for Translation and Cultural Adaptation. Value in health : the journal of the International Society for Pharmacoeconomics and Outcomes Research 2005; 8(2): 94-104.
2. Rockwood K et al. A global clinical measure of fitness and frailty in elderly people. CMAJ 2005:173;489-495.
3. Rockwood K, Theou O. Using the Clinical Frailty Scale in Allocating Scarce Health Care Resources. Can Geriatr J. 2020 Sep 1;23(3):210-215.
