# Supplementary material for: Cross-cultural adaption and inter-rater reliability of the Swedish version of the updated clinical frailty scale 2.0
Source: BMC Geriatr. 2023 Dec 5;23:803. doi: 10.1186/s12877-023-04525-6 (PMC10696827; doi:10.1186/s12877-023-04525-6)
Supplement: Supplementary file 4 — Additional file 4. GRRAS checklist. [file 12877_2023_4525_MOESM4_ESM.pdf]

GRRAS checklist for reporting of studies of reliability and agreement

Version based on Table I in: Kottner J, Audigé L, Brorson S, Donner A, Gajeweski BJ, Hróbjartsson A, Roberts C, Shoukri M, Streiner DL. Guidelines for reporting reliability and agreement studies (GRRAS) were proposed. J Clin Epidemiol. 2011;64(1):96-106

Section	Item #	Checklist item	Reported on page #
Title/Abstract	1	Identify in title or abstract that interrater/intrarater reliability or agreement was investigated.	Abstract
Introduction	2	Name and describe the diagnostic or measurement device of interest explicitly.	4
	3	Specify the subject population of interest.	7
	4	Specify the rater population of interest (if applicable).	7
	5	Describe what is already known about reliability and agreement and provide a rationale for the study (if applicable).	4-5
Methods	6	Explain how the sample size was chosen. State the determined number of raters, subjects/objects, and replicate observations.	7,9
	7	Describe the sampling method.	7
	8	Describe the measurement/rating process (e.g. time interval between repeated measurements, availability of clinical information, blinding).	7
	9	State whether measurements/ratings were conducted independently.	7
	10	Describe the statistical analysis.	8
Results	11	State the actual number of raters and subjects/objects which were included and the number of replicate observations which were conducted.	9
	12	Describe the sample characteristics of raters and subjects (e.g. training, experience).	7,9
	13	Report estimates of reliability and agreement including measures of statistical uncertainty.	9,10
Discussion	14	Discuss the practical relevance of results.	10,11
Auxiliary material	15	Provide detailed results if possible (e.g. online).	Additional file 2,3
